# The impact of smartphone use duration and posture on the prevalence of hand pain among college students

**DOI:** 10.1186/s12891-024-07685-7

**Published:** 2024-07-23

**Authors:** Faeze Dehghan Banadaki, Benyamin Rahimian, Fatemeh Moraveji, Sakineh Varmazyar

**Affiliations:** 1 Department of Occupational Health Engineering, Faculty of Health, Student Research Committee, Qazvin University of Medical Sciences, Qazvin, Iran; 2 Department of Occupational Health Engineering, Faculty of Health, Student Research Committee, Social Determinants Health Research Center and Research Institute for Prevention of Non-Communicable Diseases, Qazvin University of Medical Sciences, Qazvin, Iran

**Keywords:** Duration, Smartphone, Posture, Prevalence, Student

## Abstract

**Background:**

Excessive smartphone usage among students can lead to discomfort in their hands and fingers. This study investigates the impact of smartphone holding posture, duration of usage, and the prevalence of wrist and finger pain among university students.

**Methods:**

This cross-sectional study involved 213 university students who were selected based on inclusion criteria. Data was collected through a demographic information questionnaire. Participants self-reported five different postures for holding and interacting with a smartphone. The prevalence, frequency, severity, and interference of wrist and finger discomfort were assessed using the Cornell Hand Discomfort Questionnaires (CHDQ).

**Results:**

The study revealed that the average age of participants was 21.3 ± 2.2 years. On average, they had been using smartphones for 7.9 ± 3.1 years and spent an average of 4.9 ± 2.5 h daily holding them in their hands. In terms of discomfort, more than 25% of students reported pain in areas C (thumb finger), E (Palm Pollicis), and F (wrist) of the right hand, which was significantly related to the duration of holding the smartphone in that hand. Additionally, smartphone holding duration significantly affected areas D (palm) and F of the left hand, with over 11% of students experiencing discomfort. The most prevalent posture among students (41% of participants) involved holding the smartphone with the right hand only, with the thumb touching the screen. Notably, areas B (χ^2^ = 21.7), C (χ^2^ = 10.27), D (χ^2^ = 65.54), and E (χ^2^ = 59.49) of the right hand, as well as areas C (χ^2^ = 6.58) and E (χ^2^ = 44.28) of the left hand, exhibited significant associations with the postures of holding the smartphone.

**Conclusions:**

The duration of smartphone use and the postures in which it is held contribute to the prevalence of discomfort in the thumb area and related muscles among right-handed students.

## Introduction

Smartphones have become an integral tool in modern society, leading to their widespread use globally in recent years [1]. A study has shown that 46.3% of physiotherapy students from selected universities in Bangladesh and India were moderately addicted, and 15.3% were severely addicted to their mobile phones [2]. In Saudi Arabia, 27% of students use smartphones for 8 h daily [3].

The excessive use of smartphones is associated with thumb and wrist pain as well as wrist joint inflammation [3, 4]. A study has demonstrated that these musculoskeletal disorders stem from frequent and prolonged use of smartphone applications, which involve repetitive movements of the wrist and the interaction between the thumb and the screen [5]. Yang et al. found that adolescent students experienced increased discomfort in multiple body parts, which was associated with the duration of smartphone usage. The researchers also identified a link between smartphone use and musculoskeletal discomfort related to the duration of using ancillary functions [6]. Also, in the study, Amjad et al. reported that the duration of mobile phone usage was identified as a significant factor associated with wrist pain and disability [7].

Working with a smartphone in static and repetitive movements, coupled with improper postures, can decrease blood supply to the muscles. These issues can lead to muscle fatigue and degeneration of ligaments in areas of the body that are extensively involved in smartphone use, such as the wrist, neck, shoulders, arms, and fingers [5]. Engaging in smartphone activities without proper elbow support and frequent thumb movements can impose inappropriate static loads on the skeletal-neural and muscular structures of the upper limbs [4].

The results of Choi, W., et al.'s study showed that holding the device with the entire hand and keeping the wrist in a deviated position led to significant changes in the joint angles of the wrist, fingers and thumb. These hand posture changes can cause discomfort and musculoskeletal disorders in the hands and wrists during prolonged use of a smartphone [8].

In their study, Lee et al. also concluded that maintaining a bent or abducted position of the fingers increases muscle activity and causes significant discomfort. This unfavorable posture can lead to the development of musculoskeletal problems related to the fingers, such as fatigue and pain [9].

Excessive smartphone use can lead to enlargement of the median nerve, thumb pain, reduced grip strength, impaired hand function, and wrist pain [10, 11]. Key parameters to differentiate between minor and significant musculoskeletal disorders include pain intensity, duration of smartphone use, and frequency of symptoms [4]. In Osailan's study, a weak and negative significant relationship was found between the duration of smartphone usage and hand-grip and pinch-grip strength [12].

The hand plays a crucial role in daily activities, and with the widespread use of smartphones, particularly among students, the wrist is heavily involved in the musculoskeletal system. Improper wrist posture during smartphone use is related to the high prevalence of musculoskeletal disorders in this region [1, 3]. Therefore, this study examined the impact of duration of usage and smartphone-holding posture on hand pain prevalence in different areas among university students.

## Methods

### Participants and inclusion criteria

The study received approval from the university ethics committee under the code IR.QUMS.REC.1402.051 and contract number 28.20.24552. This cross-sectional study was conducted in 2023 and included a sample of 213 university students. The participants were randomly selected based on their willingness to cooperate and met specific the inclusion criteria. These criteria included owning a smartphone [13], sending a minimum of five emails or text messages daily [14], using a smartphone for at least two hours per day [11], having no congenital or acquired musculoskeletal abnormalities [4], not suffering from any acute or chronic musculoskeletal diseases [13], no history of hand surgery [11], no carpal tunnel syndrome [10], no presence of inflammatory arthritis [10], and no records of wrist fractures, injuries, or trauma within the past six months [15].

Out of the 221 students who expressed willingness to participate, eight individuals were excluded based on the inclusion criteria, resulting in a final sample size of 213 subjects. Three people were excluded from the study, two because they did not use a smartphone for at least two hours per day, two with a history of hand surgery, two who had experienced trauma within the past six months and one due to wrist fractures.

## Demographic questionnaire and hand posture assessment

**Fig. 1:**
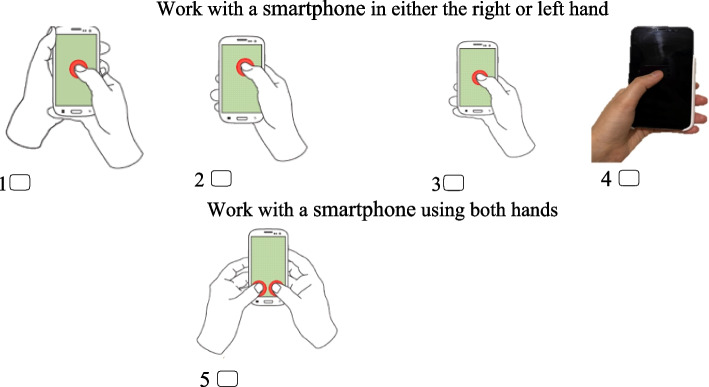
Different holding postures 1, 2,3,4, and 5 for using the smartphone. 1. Left hand to hold and right thumb to touch. 2. Right hand only to hold and thumb touching at the top of the screen (All four fingers are behind the smartphone). 3. Right hand only to hold and thumb to touch at the middle or bottom of the screen (pinky finger for support of the smartphone). 4. Left hand only to hold and thumb to touch at the middle or bottom of the screen. 5. Both hands to hold with both thumbs to touch in portrait orientation

Upon completion of the consent form, students were enrolled in the study. A demographic questionnaire was used to collect information on age, weight, height, marital status, educational level, duration of smartphone usage, and adherence to the inclusion criteria. Additionally, smartphone-holding postures were assessed through picture questions included within the demographic questionnaire (Fig. 1). The first 10 postures were as follows: 1) Left hand for holding and right hand thumb for touching; 2) Left hand for holding and right hand index finger for touching; 3) Right hand only for holding and thumb touching at the top of the screen; 4) Right hand only for holding and thumb touching at the bottom of the screen; 5) Right hand for holding and left hand thumb for touching; 6) Right hand for holding and left hand index finger for touching; 7) Left hand only for holding and thumb touching at the top of the screen; 8) Left hand only for holding and thumb touching at the bottom of the screen; 9) Both hands for holding with both thumbs touching in portrait orientation; 10) Both hands for holding with both thumbs touching in landscape orientation. However, since most students used only five postures the remaining five were removed and only those five postures were assessed (Fig. 1) [16–19].

## Cornell Hand Discomfort Questionnaires (CHDQ)

The Cornell Hand Discomfort Questionnaires (CHDQ) were used to measure the prevalence, severity, and frequency of finger, palm, and wrist discomfort in both the right and left hands. This questionnaire, which has been validated and shown reliability [20, 21], was reproduced with permission from the Human Factors and Ergonomics Laboratory at Cornell University It has been utilized in numerous studies [12, 22].

**Fig. 2:**
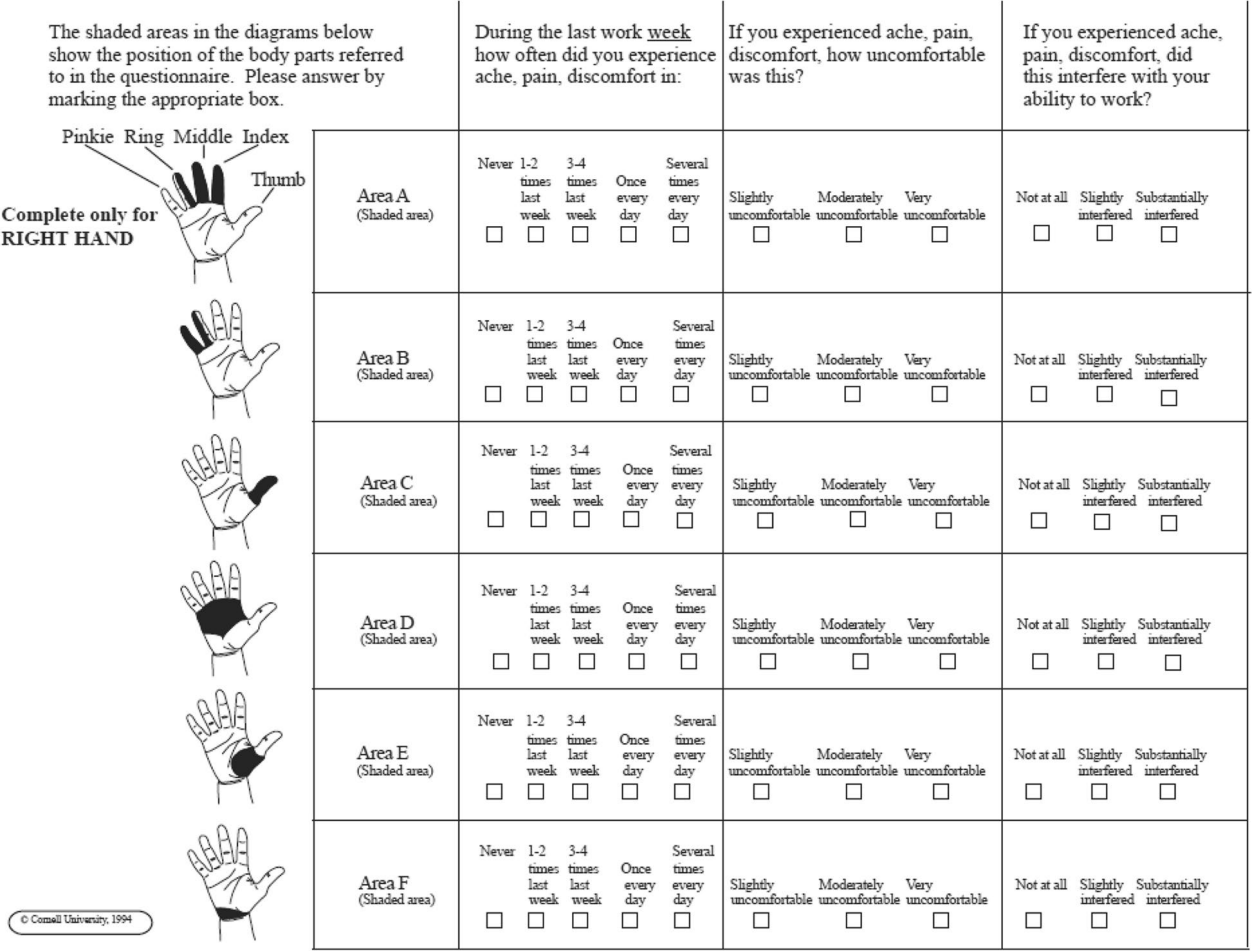
Right-hand map diagram questionnaire [21]

The CHDQ is divided into six parts for each hand. These parts include Area A (half-ring, middle, and index finger); Area B (half-ring and little fingers); Area C (thumb); Area D (palm); Area E (palm/thumb Pollicis); and Area F (wrist). Each area is associated with three specific questions: Question 1- Frequency: How often did you experience aches, pain, or discomfort during the last work week? Question 2- Severity: If you experienced aches, pain, or discomfort, how uncomfortable was it? Question 3- Interference: Did the aches, pain, or discomfort interfere with your ability to work? (Fig. 2).

The CHDQ utilizes a scoring system in which the frequency score (ranging from "never" with a score of 0 to "several times every day" with a score of 10) is multiplied by the discomfort/severity score (with values of 1 for "slightly uncomfortable", 2 for "moderately uncomfortable", and 3 for "very uncomfortable"), and further multiplied by the interference score (1 for "not at all", 2 for "slightly interfered", and 3 for "substantially interfered" (Fig. 2). By multiplying these three scores together, the total score for each part can be calculated [15]. Each part has a maximum score of 90, and the cumulative score for all six areas is 540. Higher scores indicate a greater level of discomfort [22]. To determine the prevalence of discomfort, the presence of pain in any area of the hand was considered as "yes", while the absence of pain was considered as "no". The prevalence was then evaluated based on whether ache/pain/discomfort was reported or not. The presence of pain in the hand/hands was reported as "yes" if there was discomfort in one or more areas, and "no" if there was no discomfort.

### Statistical analysis

The data analysis used the Uni-Variable logistic regression test to investigate the relationship between the variables of years and hours of smartphone use and the prevalence of discomfortin six areas on each hand. In addition, the Kruskal–Wallis test was used to analyze the relationship between total discomfort scores for each area (calculated by multiplying the scores for frequency, intensity, and interference) and different postures. The data are analyzed using SPSS-26 software.

## Results

According to the study results, the students had a mean age of 21.3 ± 2.2 years, with approximately 6 (2.8%) being married. Table 1 provides further details on the demographic and educational information of the subjects, as well as their total discomfort scores in hands.

**Table 1:** Quantitative and qualitative information of student participants in the study (*n* = 213), dominant hand, the prevalence of pain in the hand/hands, and smartphone usage patterns

**Quantitative Information**			**Qualitative Information**				
**Variable**		**Mean ± SD**	**Variable**	**Classification**		**Frequency**	**Percentage**
Age (year)		21.3 ± 2.2	Marital status	Single		207	97.2
Height (cm)		171.3±9.6		Married		6	2.8
Weight (kg)		67.4 ±13.9	Level of education	Bachelor of Science		165	77.5
Duration of smartphone use (years)		7.9 ±3.1		Master of Science		5	2.3
Holding smartphone in hand (hour)		4.9 ±2.5		Doctor of Philosophy		43	20.2
The sum of the total discomfort scoring in the right hand		35.61 ±49.87	Gender	Male		88	41.3
The sum of the total discomfort scoring in the left hand		9.20 ±21.86		Female		125	58.37
**Dominant hand, and the prevalence of pain in the hand/hands **							
**Variable**	**Classification**	**Frequency**	**Percentage**	**Variable**	**Classification**	**Frequency**	**Percentage**
The hand used to work with the Smartphone	Right	146	68.5	Prevalence of pain	No pain	72	33.8
	Left	22	10.3		Right hand	61	28.6
	Both	45	21.2		Left hand	19	8.9
-	-	-	-		Both hands	61	28.6
**Smartphone usage patterns**							
**Variable**		**Frequency**	**Percentage**	**Variable**		**Frequency**	**Percentage**
Calling by smartphone		49	23.0	Gaming /hobbies		24	11.3
Text typing /short message service		31	14.6	Training/learning		15	7.0
Social Networks		94	44.1	-		-	-

**Fig. 3:**
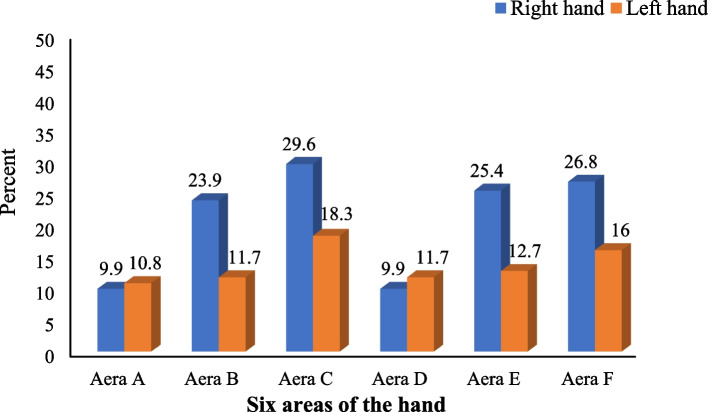
Percentage of discomfort prevalence in six areas (A-F) from the right and left hands among participating students (*n* = 213)

Figure 3 displays the prevalence of discomfort in areas of the right and left hands, including the fingers, palm, and wrist. Among the students, the highest prevalence of pain was observed in area C, which corresponds to the thumb finger, for both the right hand (29.6%) and the left hand (18.3%).

**Fig. 4:**
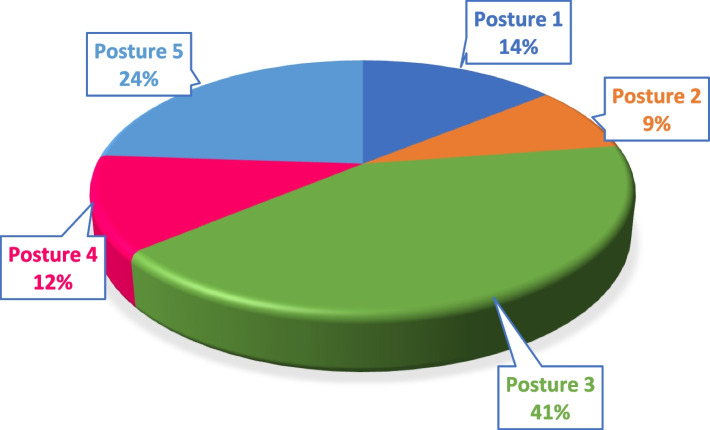
Percentage of posture types used for holding smartphones among participating students (*n* = 213)

Figure 4 reveals that the most prevalent working posture when using a smartphone with the right hand is posture three, which involves holding the smartphone in the right hand and using the thumb to operate the middle or bottom of the screen. This posture was reported by 41% of participants.

The study analyzed the relationship between the duration of smartphone use and the prevalence of discomfort in various hand areas using logistic regression. The results indicated a statistically significant association between the duration of smartphone usage (holding smartphone) and the prevalence of discomfort in the C (thumb), E (thumb Pollicis), and F (wrist) areas in the right hand. Furthermore, the study revealed a significant relationship between discomfort in the left-hand D (palm) and F (wrist) areas and hours of smartphone use (holding smartphone). However, no relationship was found between the years of using mobile phones and the prevalence of discomfort in both hands (Table 2).

**Table 2:** The results of the impact of years and hours of smartphone use on the prevalence of pain using Uni-Variable logistic regression

Risk Factors	Right-Hand					
	**Area A (CI 95%)**	**Area B (CI 95%)**	**Area C (CI 95%)**	**Area D (CI 95%)**	**Area E (CI 95%)**	**Area F (CI 95%)**
Duration of smartphone use (years)	0.93(0.80–1.08)	1.02(0.92–1.13)	1.00(0.90–1.02)	0.98(0.85–1.13)	1.03(0.93–1.14)	0.96(0.87–1.06)
Holding smartphone in hand (hour)	1.06(0.92–1.22)	1.08(0.98–1.20)	1.13(1.02–1.25)*	1.04(0.89–1.20)	1.15(1.04–1.27)**	1.11(1.01–1.23)*
**Risk Factors**	**Left-Hand**					
	**Area A (CI 95%)**	**Area B (CI 95%)**	**Area C (CI 95%)**	**Area D (CI 95%)**	**Area E (CI 95%)**	**Area F (CI 95%)**
Duration of smartphone use (years)	1.04(0.90–1.19)	1.06(0.93–1.22)	1.06(0.94–1.19)	1.07(0.93–1.23)	1.03(0.90–1.17)	1.05(0.93–1.18)
Holding smartphone in hand (hour)	1.03(0.89–1.18)	1.11(0.98–1.26)	1.03(0.92–1.15)	1.16(1.02–1.31)*	1.00(0.87–1.15)	1.13(1.01–1.26)*

^*^p ≤ 0.05

^**^ *p* ≤ 0.001

Significant disparities were found in total discomfort scores in areas B, C, D, and E of the right hand when different smartphone holding postures were used. In addition, significant differences were observed in total discomfort scores in areas C and E of the left hand when various smartphone holding postures were used.(Table 3).

**Table 3:** The relationship between holding a smartphone in the hands and experiencing discomfort in different areas of the hands using the Kruskal Wallis test

Variable Independent	Posture 1,2,3,5 (Right hand or both hands)		Posture 1,4,5 (Left hand or both hands)	
Variable Dependent				
	Chi-square	*P*	Chi-square	*P*
Area A	2.35	0.56	0.55	0.97
Area B	21.7	0.00^**^	5.41	0.06
Area C	10.27	0.01^*^	6.58	0.03^*^
Area D	65.54	0.00^**^	0.26	0.87
Area E	59.49	0.00^**^	44.28	0.00^**^
Area F	0.56	0.90	0.01	0.99

^*^p ≤ 0.05

^**^ *p* ≤ 0.001

As shown in Table 4, the prevalence of pain is influenced by the dominant hand used when using a smartphone, while gender and smartphone usage patterns did not impact the discomfort experienced in the hand or hands.

**Table 4:** The relationship between the prevalence of hand pain and factors such as gender, dominant hand, and type of smartphone use was analyzed using the chi-square test

Variable Independent	Gender			Dominant hand			Smartphone usage patterns		
Variable Dependent									
Prevalence of hand/hands pain	χ^2^	df	P	χ^2^	df	P	χ^2^	df	P
	7.02	3	0.07	21.78	6	0.001**	4.64	12	0.96

^*^p ≤ 0.05

^**^p ≤ 0.001

## Discussion

### Relationship between years and hours of smartphone use with the prevalence of discomfort in right and left hands

The study did not find a significant relationship between the number of years of mobile phone use and the prevalence of hand pain. Students may have owned phones for many years but used them less due to being less user-friendly and having fewer capabilities. However, in recent years, with the availability of higher-end phone models and the impact of the coronavirus disease, students have been using smartphones for more hours.

The results of the univariate analysis (Table 2) indicated a significant relationship between the variable of hours of smartphone use and the prevalence of pain in areas C (thumb finger), E (thumb Pollicis), and F (wrist) of the right hand, as well as areas D (palm) and F (wrist) of the left hand. This shows that prolonged smartphone use is associated with an increased prevalence of pain in these regions. As indicated in Fig. 3, a quarter to a third of students in areas C, E, and F reported experiencing discomfort in their right hand (range 25.4%-29.6%), and more than 10% reported discomfort in areas D and F of the left hand. The higher prevalence of pain in the right-hand area could be attributed to the higher percentage of students (68.5%) who use smartphones predominantly with their right hand. Abdulahi's study, conducted among medical students, reported that 31% of participants experienced pain in the hand/wrist. The study also found a significant association between the duration of smartphone use and this factor. These findings are consistent with the results of the present study[23]. Similarly, Amjad's results indicated a relationship between the duration of mobile phone use and wrist pain, as well as an increased likelihood of experiencing pain in the right hand [7].

When sending text messages, a significant amount of force is exerted by the thumb on the smartphone keyboard [12]. As shown in Fig. 4, 64% of participants use their right thumb for typing messages or operating the phone (postures 1, 2, and 3). This can lead to discomfort in the thumb area and the Pollicis muscles of the right hand. Furthermore, holding the smartphone for extended periods and maintaining a static position increases the physical load on the hand joints over time, eventually causing discomfort [12, 22, 24]. The repetitive movements when rapidly typing or performing other phone-related activities can strain muscles in the thumb region such as the adductor Pollicis, flexor Pollicis Brevis, abductor Pollicis Brevis, and Opponens Pollicis as well as put pressure on the wrist. This, in turn, can worsen muscle pain in the thumb area and hands [12, 22, 25, 26].

The muscles in the palm are heavily used when holding a smartphone for texting or phone usage [12]. The frequent pressure from the lower right corner of the smartphone on the palm (D area) or the thumb Pollicis (E area) muscles can cause discomfort in these areas. Furthermore, this localized pressure can affect the ulnar nerve in the palm, leading to discomfort in the wrist area (F area) [27].

Based on the findings of Table 4, there is no significant relationship between the prevalence of hand pain and smartphone applications. However, a notable relationship exists between the prevalence of pain and the dominant hand. This indicates that students who use their right, left, or both dominant hands have a higher prevalence of pain in their corresponding hands. The study by Sharan et al. suggests that individuals who favor their dominant hand tend to hold their mobile phones in that hand, impacting the prevalence of musculoskeletal disorders in various hand regions [28].

In Gustaffson et al.'s study [29], they demonstrated that sending text messages on mobile phones and repeated thumb movements have short-term and long-term effects on the prevalence of musculoskeletal disorders in the upper limbs. Similarly, Sharl et al.'s [30] study highlights the occurrence of musculoskeletal disorders in the hand among individuals who heavily use mobile phones, aligning with the present study's findings.

Periyar et al.'s [22] study revealed that prolonged cell phone usage is associated with musculoskeletal issues in the neck and hand, particularly the thumb, resulting in short-term complications and long-term disability. Furthermore, the findings from studies conducted by Hua, B. et al. [24]. and Sohel Ahmed et al.[2] indicate that the shaded areas D and F, corresponding to both hands, are linked to smartphone addiction, aligning with the outcomes of the current study [24]. Ayman Baabdulla's [31] study concluded that students who extensively use smartphones experience pain in their thumbs and wrists, which aligns with the present study's findings.

## Relationship between smartphone-holding posture with the prevalence of discomfort in right and left hands

When using smartphones, it is common for individuals to hold the device with one or two hands and use their thumb to interact with the screen [32]. Three of the most common ways of typing and holding a mobile phone are: using both hands for holding and touching (typing with two thumb fingers), using only the right or left hand for holding and touching (typing with the same hand finger), and using one hand for holding and the opposite hand for touching (typing with another hand finger) [25].

In this study, the results from Table 3, analyzed using the Kruskal–Wallis test, indicated a significant association between the holding smartphone postures (two-finger and one-finger typing) and discomfort in areas B, C, D, and E of the right hand (*P* ≤ 0.01). Furthermore, a significant relationship was observed between areas C and E of the left hand (*P* < 0.05).

Fifty percent of the participants used postures 2 and 3 of the right hand (Fig. 4) when holding a smartphone. In these postures, the palm and little finger support the lower part of the smartphone. These hand positions are lined to significant discomfort in areas B and D of the right hand (*P* ≤ 0.01) [19, 24, 33].

The way a smartphone is held, whether with one hand or two hands, affects the position of the thumb [34]. Studies show that using a smartphone with one hand leads to more musculoskeletal symptoms in the shoulder-arm and thumb areas [32], while holding it with two hands causes muscle tension in the hand, resulting in discomfort in the thumb and wrist [16]. According to Table 3, both two-finger and one-finger postures of holding the phone significantly cause discomfort in the thumb (areas C, E) of both the right and left hands (*P* < 0.05).

The findings of the study conducted by Jung SI and colleagues [35] align with the results of the present study regarding the significance of discomfort in the thumb area of both the left and right hands.

The findings of Trudeau MB et al.'s [36] study indicate that holding the phone with two hands resulted in discomfort in the thumb and wrist. However, these findings differ from the present study, which observed wrist discomfort. This discrepancy might be due to the use of different questionnaires to assess discomfort in the hand areas. Furthermore, it is worth noting that the current study only examined two of the five postures related to holding the mobile phone with both hands and did not investigate them separately.

The findings of the present study highlight that musculoskeletal injuries in the hand are often caused by repetitive strain and cumulative trauma. Consequently, it is recommended that students reduce their smartphone usage time and minimize holding it in their hands. The use of auxiliary tools like a smartphone stand can be beneficial. Taking frequent breaks during smartphone use and incorporating a 20-min interval after prolonged usage is also advised to prevent effects and mitigate the risk of pain. Given the significance of the thumb in smartphone usage, users are encouraged to employ alternative fingers for typing, reducing strain on the thumb [2]. Additionally, voice-to-text software can be an effective alternative to typing, further reducing the strain on the hand.

This study will be beneficial for students, especially young individuals who frequently use smartphones. It provides important insights into the increasingly harmful consequences of long-term smartphone usage. By raising awareness about these effects, the study can help students make informed decisions and take necessary steps to maintain a healthy balance in their smartphone usage.

## Conclusion

The most common posture used by students (two-fifths) is holding the smartphone with the right hand with the thumb touching the middle or bottom of the screen. Additionally, the most reported pain (one-third to one-quarter) was in area C (thumb finger) and area E (Pollicis muscle of thumb) of the right hand. The prevalence of discomfort in both areas C and E of both hands was significantly associated with the postures used while holding smartphones. Moreover, the duration of daily smartphone holding significantly impacts discomfort in areas C, E, and F (wrist) of the right hand and areas D and F of the left hand. Furthermore, there is a significant relationship between the dominant hand and the prevalence of musculoskeletal disorders. Therefore users should try to use both hands when holding the smartphone whenever possible. Using the non-dominant hand for holding the smartphone can help reduce the risk of skeletal disorders.

### Limitations and suggestions

One limitation of the current study is the lack of investigation into the impact of smartphone dimensions and weight on hand/wrist discomfort. It is recommended that future studies consider evaluating the angles of the fingers, especially the thumb, in various postures, as well as the factors mentioned above. Another limitation is hand size which can affect the prevalence of hand pain. This implies that future research should focus on this factor.

## Data Availability

The data in this article will be shared on reasonable request to the corresponding author.
